# Cognition before and after psychosis onset: A naturalistic study of change, heterogeneity, and prognosis^☆^

**DOI:** 10.1016/j.scog.2025.100387

**Published:** 2025-08-29

**Authors:** Maria Lee, Alexis E. Cullen, Granville J. Matheson, Zheng-An Lu, Sarah E. Bergen, Carl M. Sellgren, Sophie Erhardt, Helena Fatouros-Bergman, Simon Cervenka

**Affiliations:** a Centre for Psychiatry Research, Department of Clinical Neuroscience, Karolinska Institutet, & Stockholm Health Care Services, Region Stockholm, Sweden; b Division of Insurance Medicine, Department of Clinical Neuroscience, Karolinska Institutet, Stockholm, Sweden; c Department of Psychosis Studies, Institute of Psychiatry, Psychology & Neuroscience, King's College London, London, UK; d Department of Psychiatry, Columbia University, New York, 10032, NY, USA; e Department of Biostatistics, Columbia University Mailman School of Public Health, New York, 10032, NY, USA; f Department of Medical Epidemiology and Biostatistics, Karolinska Institutet, Stockholm, Sweden; g Department of Physiology and Pharmacology, Karolinska Institutet, Stockholm, Sweden; h Department of Medical sciences, Psychiatry, Uppsala University, Uppsala, Sweden

**Keywords:** Cognition, Schizophrenia, First-episode psychosis, Longitudinal, Trajectories, C4A, Polygenic risk scores

## Abstract

**Aims:**

Cognitive dysfunction is a core feature of psychotic disorders. The degree of impairment varies greatly between individuals, which may reflect different levels of decline from pre-morbid functioning. Diverse trajectories of cognitive change prior to or during development of psychosis have been hypothesized to reflect distinct underlying pathological processes. Our primary aim was to model cognitive change over time in a sample of individuals with first-episode psychosis (FEP) and controls. The secondary aim was to explore associations between cognitive change, clinical outcomes and select biological markers.

**Methods:**

Our sample consisted of 72 individuals with FEP and 53 controls. School grades from nationwide population registers were used as a proxy for pre-morbid cognitive ability. All participants underwent formal cognitive testing at psychosis onset, with a subset returning for testing at 1,5 year follow up. Cognitive change was modelled using linear mixed-effects models, and resulting change scores were correlated to polygenic risk scores, cerebrospinal fluid levels of complement protein C4A and clinical outcomes.

**Results:**

Groups did not differ in school performance prior to psychosis. Psychosis onset was associated with marked cognitive decline in FEP individuals, who subsequently performed significantly worse than controls. However, cognitive change over time varied widely between FEP individuals. Degree of cognitive change was not associated with the selected biological variables but did predict worse clinical outcomes.

**Conclusions:**

Individual cognitive trajectories may be a clinically relevant topic for further study, and larger studies are needed to further explore their potential role in stratified models of care.

## 1 Introduction

Cognitive impairment is common in psychotic disorders such as schizophrenia (Harvey et al., 2022; Schaefer et al., 2013; Mesholam-Gately et al., 2009), and has been related to functional impairment both at illness onset (Kharawala et al., 2022; Allott et al., 2011) and in later phases of the disorder (Kharawala et al., 2022; Halverson et al., 2019; Green et al., 2004). Cognitive problems tend to emerge before the onset of positive and negative psychotic symptoms, with mild cognitive deficits evident already in childhood (McCutcheon et al., 2023; Fett et al., 2022; Dickson et al., 2012). How cognition develops in the years leading up to psychosis, and whether this differs between individuals, are questions of great importance, as this knowledge may provide insights into underlying pathological processes.

Cognitive change is often studied by estimating pre-morbid cognitive ability using reading tests, such as the National Adult Reading Test (NART) (Nelson and Willison, 1991), where single irregular words are read aloud (Hochberger et al., 2020). Investigations using reading tests combined with demographic factors find that a large proportion of patients with psychosis perform at a level significantly below their predicted premorbid abilities (Hochberger et al., 2020; Keefe et al., 2005). However, these tests are not available in all contexts and ultimately, due to their retrospective nature, only provide estimates of premorbid ability.

In clinical practice, pre-morbid cognitive ability is more commonly assessed using school grades and academic achievements, as these are readily accessible. School grades do not directly correspond to results from cognitive tests, as scholastic achievement is also influenced by other factors such as socioeconomic status and personality traits, including self-efficacy (Boman, 2023). Yet, cognitive ability has consistently been found to be the strongest predictor of scholastic achievement (Boman, 2023; Roth et al., 2015; Deary et al., 2007) with correlation coefficients of up to 0.7 reported (Roth et al., 2015). A recent meta-analysis found that general academic achievement before age 16 was significantly lower in those who went on to develop schizophrenia compared to those who did not, although effect sizes were small (Cohen's *d* = -0.29) (Dickson et al., 2020). Some follow-back studies have demonstrated that individuals who go on to develop psychotic disorders display a drop in academic performance in the ages 13-16 (Fuller et al., 2002), as well as a widening gap compared to healthy individuals over time (Bilder et al., 2006). However, there is a lack of research on the relationship between prior scholastic achievement and cognitive function after illness onset, as well as how these trajectories relate to biological markers and clinical outcomes.

Furthermore, cognitive performance in psychotic disorders differs greatly between individuals (Carruthers et al., 2019), and this inter-individual variability is significantly greater in patients than in healthy controls (Lee et al., 2024). Whether variability at illness onset reflects differences in trajectories rather than different pre-morbid levels of cognition is poorly understood. Factors influencing the longitudinal course of cognition may differ from those associated with cognitive performance at psychosis onset. Prior studies in this area have primarily relied on reading tests, showing that the degree of deviation from expected pre-morbid ability is related to functional outcome and neurophysiological measures (Hochberger et al., 2020). While such studies suggest that cognitive trajectories are associated with treatment response and other outcomes, it is important to acknowledge that they did not employ longitudinal measurements. Cohort studies with longitudinal data have used the degree of cognitive change to predict risk of developing schizophrenia (Osler et al., 2007; MacCabe et al., 2013; Reichenberg et al., 2005; Jones et al., 1994), however, to our knowledge, inter-individual variability in cognitive change has not been quantified previously.

A vast number of biological factors may impact cognitive change. Genetic characteristics are known to be the most important risk factor for psychotic disorders (Trubetskoy et al., 2022), and higher polygenic risk scores (PRS) have previously been linked to cognitive trajectory groups with low premorbid functioning and steeper decline (Dickinson et al., 2020). Furthermore, genetic studies have implicated potential biological mechanisms involved in the pathophysiology of psychotic disorders. One such mechanism involves the complement system - specifically the immune signaling protein complement component 4A (C4A) - which has been suggested to promote excessive synaptic pruning (Trubetskoy et al., 2022; Sekar et al., 2016). Synaptic pruning is a developmental process that occurs during the same time frame as the typical prodromal phase, and may hence impact cognitive trajectories.

In the present study we sought to extend prior research by using longitudinal data acquired before individuals developed their first psychotic episode. Using a well-characterized cohort of FEP individuals, our aims were to examine the between-individual variability in the magnitude of cognitive change prior to psychosis onset, and to relate the degree of cognitive change to subsequent health care usage (days in hospital and number of hospital admissions). These variables have previously been linked to cognitive function (Kadakia et al., 2022) but, to our knowledge, have not been examined in relation to cognitive change before psychosis onset. A further aim was to explore potential relationships between the degree of cognitive change and levels of C4A, as well as PRS for schizophrenia and intelligence.

## 2 Methods

### 2.1 Participants

Individuals with FEP were recruited through the ongoing Karolinska Schizophrenia Project (KaSP), which enrolls patients who experience their first psychotic episode and who are either antipsychotic-naïve or within four weeks of first exposure to antipsychotic medication. The main inclusion criterion for patients was a diagnosis of psychotic disorder (schizophrenia, schizophreniform disorder, schizoaffective disorder, delusional disorder, brief psychotic disorder and psychotic disorder not otherwise specified) according to Structured Clinical Interview for DSM-IV (SCID) (First and Gibbon, 2004), conducted by a psychiatrist or clinical psychologist. Controls were recruited through advertisement. Exclusion criteria for all participants were: severe somatic illness or neurological disorder and current abuse of alcohol or illegal drugs (including cannabis) or a history of such abuse. Further exclusion criteria for controls were current psychiatric illness assessed by Mini International Neuropsychiatric Interview (MINI) (Sheehan et al., 1998), lifetime use of antipsychotics or having a first-degree relative with psychotic illness. The study was approved by the Regional Ethics Committee in Stockholm (diary number: 2010/879-31-1) and all participants provided written informed consent.

For the present study, participants were included if they had data from at least one administration of the MATRICS Consensus Cognitive Battery (MCCB) and registry data on school grades from at least one time-point. The final analytic sample consisted of 72 individuals with FEP and 53 controls.

### 2.2 Data collection

As part of KaSP, individuals with FEP undergo clinical characterization, including assessment with the Positive and Negative Syndrome Scale (SCI-PANSS) (Kay et al., 1987), Global Assessment of Function (GAF) (Jones et al., 1995), and the Clinical Global Impression Scale (CGI) (Busner and Targum, 2007)). All participants in the study underwent cognitive testing with the MCCB (Nuechterlein et al., 2008), as well as lumbar puncture and blood sampling. The MCCB is the gold standard cognitive battery in schizophrenia research and shows high correlations with other measures of general intelligence, such as the Wechsler Abbreviated Scale of Intelligence Estimated Full Scale IQ (August et al., 2012). It consists of 10 subtests, measuring seven domains: Processing Speed, Working Memory, Verbal Learning, Visual Learning, Reasoning/Problem-solving, Attention and Social Cognition. For this study, we selected the global neurocognitive composite as our outcome measure, as it was considered to better correspond to general academic achievement. This measure includes nine out of the 10 subtests, excluding the social cognitive domain. Cognitive test scores were derived using the MCCB norms, with corrections applied for age and sex. American normative data were used, which have been shown to be applicable in Scandinavian populations (Mohn et al., 2012). Cognitive testing at baseline represents timepoint 3 in subsequent statistical models. All participants were invited back for 1,5 year follow-up assessments, where clinical interviews and cognitive testing was repeated, and cognitive scores from follow-up were used as timepoint 4.

### 2.3 School grades

The Swedish National Agency for Education provides individual-level data on school grades (The Swedish National Agency for Education, 2024). Data were available from year 9 (ages 15-16 years), which marks the end of compulsory school in Sweden and from year 12 (ages 18-19 years), which is the end of upper secondary school. Grades from year 9 represents timepoint 1 in subsequent models, and grades from year 12 represent timepoint 2. Approximately 75-80 % of students who complete year 9 also complete year 12 (based on population level statistics).

### 2.4 Clinical outcomes using population registries

Health care use and medication use information was available for participants via the National Patient Register (NPR) (The Swedish National Board of Health and Welfare, 2024a) and the Swedish National Prescribed Drug Register (PDR) (The Swedish National Board of Health and Welfare, 2024b), from time of inclusion in study until 2022-09-30 (see Supplementary Methods for information about the Registries). Clinical outcomes were defined using a range of registry variables. For inpatient health care use, we calculated the number of hospital days (excluding mandated involuntary care in outpatient settings, “öppen psykiatrisk tvångsvård” in Swedish) and the number of hospitalizations due to any psychiatric diagnosis or self-harm (see Supplementary Material for exact definition) over the entire follow-up period. To capture the need for continued psychiatric care and anti-psychotic medication following the first psychotic episode, we chose to focus on long-term clinical variables: 1) outpatient psychiatric care in the fifth year after inclusion in study and 2) dispensation of antipsychotic medication during the same time period. This was determined by outpatient visit in psychiatric care (12-month period prevalence prior to the 5-year mark of inclusion in study) with a main diagnosis for (a) any psychiatric diagnosis, (defined as International Classification of Diseases (World Health Organization, 2004) (ICD) codes F10-F19, F30-F99), and (b) a main diagnosis corresponding to schizophrenia spectrum and other psychotic disorders (ICD codes F20 to F29). The same principle was applied for dispensation of antipsychotic medications (not including mood-stabilizers, for a full list see Supplementary Material).

### 2.5 Polygenic risk scores

We hypothesized that more severe decline would be related to higher PRS for schizophrenia and lower PRS for intelligence. PLINK version 1.90 was employed to perform quality control of genetic data and generate PRSs (Chang et al., 2015). The base datasets for schizophrenia and intelligence PRSs are the summary statistics for the largest published GWAS (Trubetskoy et al., 2022; Savage et al., 2018) for these phenotypes. The target dataset for PRS calculation is the genotype data (provided using the InfiniumPsychArray-24 v1.1 BeadChip) from the study sample consisting of 93 individuals. The SNPs were excluded if they: (1) showed deviations from Hardy-Weinberg equilibrium (*P* < 1 × 10^-6^) or; (2) had a genotype missing rate >5 %; or (3) had a minor allele frequency < 1 %, leaving a total of 318, 915 SNPs. Individuals with a genotype missing rate >5 % (*N* = 3) were excluded.

Quality control for the base datasets was performed by removing multiallelic and duplicated SNPs. After that, the target and base datasets were harmonized by extracting SNPs that were included in both datasets and aligning the effect alleles. After clumping the SNPs at r^2^ < 0.1 within 250 kb, a total of 58,829 and 54,281 SNPs were included as valid predictors for the schizophrenia and intelligence PRSs, respectively.

The PRSs were generated using the PRS-principal component analysis (PCA) approach (Coombes et al., 2020). First, the PRS at 10 different *p*-value thresholds (5e-8, 1e-6, 1e-4, 0.001, 0.01, 0.05, 0.1, 0.2, 0.5, 1) were calculated. Next, PCA was applied to PRSs at all thresholds. The standardized first components derived from PCA were utilized as the final PRSs tested for associations with cognitive change. This consolidates risk from the individual PRSs, resulting in a single measure that is more powerful than a PRS at any specific *p*-value threshold and eliminating the need for multiple tests across thresholds.

### 2.6 C4A protein

Our hypothesis was that higher C4A levels would be associated with more severe cognitive decline. C4A protein levels were measured in cerebrospinal fluid (CSF) using targeted mass spectrometry and the Micro BCA Protein Assay Kit. Measurements were performed in a 96-well plate and fluorescence read by plate reader FLUOstar Omega. Sample preparation was performed on the Agilent AssayMAP Bravo Platform. Further details regarding sample collection and data analysis have been published previously (Gracias et al., 2022).

### 2.7 Statistical analysis

Individual participant grades were converted to percentiles by comparing each performance to percentile values for the entire population that graduated in Sweden that year. This was done separately for grades from year 9 and year 12. The resulting percentile scores were then transformed to their corresponding z-scores using a one-to-one independent transformation which maps the scores from a bounded uniform to a normal distribution. This transformation more accurately represents distances between extreme values, and allows the use of parametric statistical models (for details of this procedure and flow chart see Supplementary Material).

For the MCCB, one FEP individual was missing results for one of the 9 subtests used to derive the neurocognitive composite score. Imputation of this value was performed via stochastic regression using the mice package (van Buuren and Groothuis-Oudshoorn, 2011), in R, following the recommended methods described in the MCCB manual (Nuechterlein and Green, 2006). The MCCB software produces percentile values, which were converted to z-scores in the same 1 to 1 sample independent transformation as for grades.

Linear mixed-effects modelling (LME) was used to explore cognitive trajectories using data across all four time-points. Models were built in consecutive steps, starting with random intercept for all participants, then adding, in order: 1) timepoint, 2) group (FEP individual or control) and 3) timepoint*group interaction as predictor variables. Cognitive change scores were estimated for each individual as random slopes from their pre-morbid (timepoint 1 and 2) to post-morbid (timepoint 3 and 4) measurements. Akaike Information Criterion (AIC) and Bayesian Information Criterion (BIC) values were used to compare model fit at each step as model complexity increased. See Supplementary methods for details on full LME model specification and quality check.

To specifically assess premorbid-to-postmorbid changes in FEP individuals, we rearranged the dummy fixed effect variables to estimate the combined effect of becoming unwell (i.e. timepoints 1 and 2 (school grades) to timepoints 3 and 4 (cognitive testing after psychosis)). Because this alternative model is identical in all respects except for the dummy variable construction, it yields identical predicted values and residuals but allows for interpretation of this combined effect.

The relationship between cognitive change and C4A was explored using multiple linear regression, with age and cognitive change entered as predictors. Simple linear regression was used to analyze the relationship between cognitive change and PRS scores.

Cognitive change was also evaluated as a predictor of several aspects of clinical outcome. Count variables (number of hospital days and number of hospital admissions) were characterized by excess zeroes and were therefore analyzed using hurdle models (Feng, 2021). These models allow for simultaneous analysis of the likelihood of having any admissions/hospital days (in the binary component of the model) and the number of admissions/hospital days among those who experienced at least 1 admission/hospital day (truncated count component) (Min and Agresti, 2005). The choice of statistical model was guided by distribution of data and model fit (assessed using AIC). Logistic regression was used for the binary component of the models and truncated negative binomial models (AIC for truncated Poisson = 9489, AIC for truncated negative binomial = 714) were employed for the count component of the model.

To explore potential relationships between the degree of cognitive change and long-term likelihood of psychiatric care utilization, we used logistic regression models to examine three outcomes: 1) outpatient visits with any psychiatric diagnosis, 2) visits with a main diagnosis of a schizophrenia spectrum disorder, and 3) dispensation of antipsychotic medications.

The statistical analysis plan was pre-registered on osf.io. All statistical analyses were conducted in R (Team, 2022) version 4.4.0 (2024-04-24). LME models were calculated using the lme4 (Bates et al., 2015) and lmerTest packages (Kuznetsova et al., 2017), and hurdle models using the pscl package (Zeileis et al., 2008). Given the exploratory nature of the study, no corrections for multiple comparisons were applied.

## 3 Results

Demographic information is provided for the final sample of 72 FEP individuals and 53 controls in Table 1.

**Table 1:** Demographic comparison of study participants.

	Baseline					1.5 year follow up				
	FEP	HC	Chi-2	t	p	FEP	HC	t	Chi-2	p
N	72	53				30	23			
Age (SD)	26.6 (6.0)	26.3 (5.1)		−0.34	0.737	29.0 (6.5)	27.9 (6.2)	−0.65		0.516
Sex (female) (%)	26 (36)	29 (55)	3.57		0.059	12 (40)	13 (57)		0.84	0.360
Years of education (SD)	13.9 (2.8)	14.7 (2.1)		1.83	0.071	13.4 (2.5)	15.9 (2.5)	3.55		0.001⁎

FEP = Individual with First episode psychosis, HC = healthy control participant, SD = Standard deviation.

⁎ p < 0.05.

The two groups were of similar age at inclusion and there were no statistically significant differences in sex composition or years of education at baseline, although there was a trend towards higher proportion of females and more years of education in the controls. At follow-up, there was a significant difference in years of education between groups (with FEP participants completing fewer years of education on average) but not for age or sex. Clinical data for individuals with FEP at the baseline assessment and the 1.5-year follow-up can be found in Table 2.

**Table 2:** Clinical data for individuals with FEP at baseline and follow-up.

	Baseline	1.5-year follow-up
N	72	30
Diagnosis, N (%)	25 (35) Schizophreniform disorder 20 (28) Schizophrenia 17 (24) Psychotic disorder NOS 4 (5) Delusional disorder 3 (4) Brief psychotic disorder 2 (3) Schizoaffective disorder 1 (1) Severe depressive episode with psychotic features	0 (0) Schizophreniform disorder 16 (53) Schizophrenia 5 (17) Psychotic disorder NOS 1 (3) Delusional disorder 1 (3) Brief psychotic disorder 2 (7) Schizoaffective disorder 0 (0) Severe depressive episode with psychotic features 1 (3) Depressive episode, no psychosis 4 (13) No remaining psychiatric diagnosis
PANSS total, mean (SD)	71.4 (20)	53.5 (13.9)
PANSS positive, mean (SD)	18.3 (6)	11.6 (3.8)
PANSS negative, mean (SD)	16.6 (7)	14.3 (6.2)
PANSS general, mean (SD)	36.5 (11)	27.7 (7.5)
GAF, mean (SD)	43.3 (14)	63.9 (19.1)
CGI-S, mean (SD)	4.4 (1)	2.8 (1.4)

PANSS = Positive and Negative Syndrome Scale, GAF = Global Assessment of Function, CGI-S = Clinical Global Impressions Scale.

**Fig. 1:**
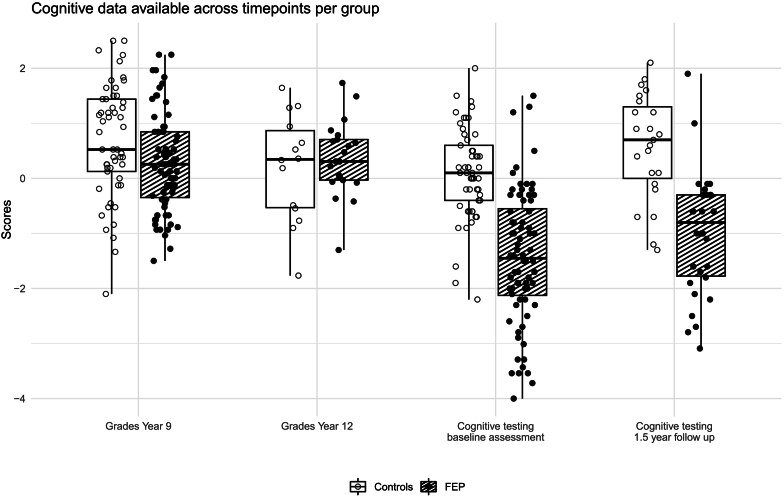
Cognitive data available across timepoints per group.

School grade data from year 9 (timepoint 1) were available for 71 patients and all controls, while year 12 grades (timepoint 2) were available for 21 patients and 14 controls. Baseline cognitive testing data (timepoint 3) were obtained from all 72 patients and 53 controls. At the 1.5-year follow-up (timepoint 4), cognitive data were available for 30 patients and 23 controls. Fig. 1 shows the available information using boxplots with individual data points superimposed.

LME models were used to investigate cognitive function over time, defined as either school grades or results from cognitive tests. As the time interval between completion of grade 9 and study inclusion varied considerably (see Supplementary Material, Table 1), time was coded categorically according to the four assessment phases (1 through 4). Adding random slopes for patients, to model individual cognitive change from premorbid (school grades) to postmorbid (cognitive testing after illness onset) timepoints, significantly improved model fit (χ2(1) = 35.59, *p* < 0.001, AIC/BIC updated model: 856/898). Adding random slopes in a similar manner for healthy controls further improved model fit (χ2(1) = 12.76, p < 0.001, AIC/BIC updated model: 845/891) (See Supplementary Material for the final LME model specified). A summary of the final model, including random intercept, timepoint, group, timepoint*group interaction and random slopes for patients and controls, can be found in (Table 3).

**Table 3:** Summary of final LME model of cognitive change across timepoints.

Fixed effects	Estimate	SE	p-value
Intercept	0.676	0.120	<0.0001^⁎⁎⁎^
Timepoint 2	−0.445	0.154	0.005^⁎⁎^
Timepoint 3	−0.584	0.125	<0.0001^⁎⁎⁎^
Timepoint 4	−0.204	0.155	0.192
Group: FEP	−0.401	0.158	0.013^⁎^
Time by Group, Timepoint 2: FEP	0.321	0.201	0.115
Time by Group, Timepoint 3: FEP	−1.127	0.182	<0.0001^⁎⁎⁎^
Time by Group, Timepoint 4: FEP	−1.240	0.221	<0.0001^⁎⁎⁎^


Random effects (standard deviations)	SD estimate		
Intercept	0.754	–	–
Change timepoint 1-2 to timepoint 3-4 FEP	0.934	–	–
Change timepoint 1-2 to timepoint 3-4 controls	0.665	–	–
Residual variance	0.442	–	–

**Fig. 2:**
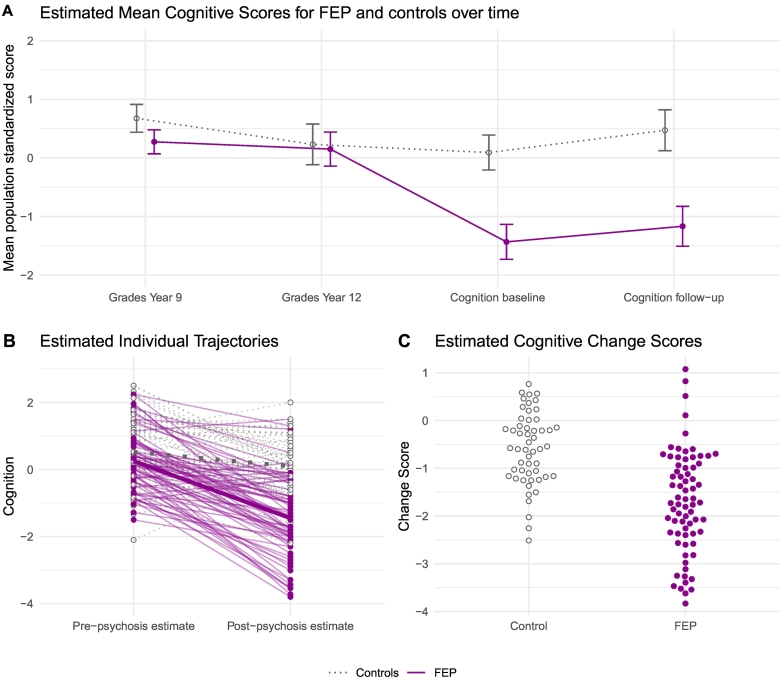
Linear Mixed Effect model estimates. Panel A: Marginal means plot of estimated mean cognitive scores for individuals with FEP and controls at the four timepoints. Panel B: Estimated individual trajectories in cognitive change from before to after psychosis onset, with median change per group in thick lines. Panel C: Estimated cognitive change scores in FEP and controls. FEP depicted by solid circles and solid lines, controls in open circles and dashed lines.

Table 4 presents model-estimated means for each timepoint and contrasts between timepoints for both groups,; these results are also depicted graphically in Fig. 2A. Among individuals with FEP, there was a significant decline between timepoint 2 and timepoint 3, with illness onset associated with an average loss of 1.12 z-scores: over 1 standard deviation. For controls, there was a significant drop between timepoint 1 and 2, but no significant difference from 2 to 3. Controls significantly improved from baseline cognitive assessment to 1.5-year follow-up, whereas FEP individuals did not.

**Table 4:** Model estimated mean cognitive scores per timepoint and contrasts between timepoints.

	Individuals with FEP			Controls		
Mean cognitive score	Estimate	SE	95 % CI	Estimate	SE	95 % CI
Timepoint 1	0.275	0.10	0.07–0.48	0.676	0.12	0.44–0.91
Timepoint 2	0.151	0.15	−0.14–0.44	0.230	0.18	−0.12–0.58
Timepoint 3	−1.433	0.15	−1.73 – −1.14	0.092	0.15	−0.21–0.39
Timepoint 4	−1.167	0.17	−1.51 – −0.83	0.472	0.18	0.12–0.82


Contrasts	Estimate	SE	p	Estimate	SE	p
Time2 – Time1	−0.124	0.13	0.917	−0.445	0.16	0.031
Time3 – Time2	−1.584	0.17	<0.0001	−0.138	0.18	0.971
Time4 – Time3	0.266	0.11	0.113	0.380	0.13	0.021

In terms of inter-individual variability, the modelled variability estimate for the random slopes was greater in individuals with FEP compared to controls (SD estimate 0.934 vs. 0.665), visualized in Fig. 2B-C. The mean effect of becoming unwell in the FEP group was −1.12 (95 % CI: −1.48 to −0.77), and the estimated 95 % range at the individual level (i.e. after incorporating the random slope variance) was between −2.95 to 0.70.

### 3.1 Clinical outcomes

During the study period (from inclusion until 2022-09-30) 79 % of patients were admitted to inpatient care at least once due to a psychiatric diagnosis or self-harm. Among those that were ever hospitalized, the mean number of hospitalizations was 2.8 (median = 2, range = 1-13). The mean number of hospital days (excluding days in mandated involuntary care in outpatient settings) for patients who were ever admitted to inpatient care was 98.2 (median = 40, range 3–759). Descriptive statistics and results for the hurdle models are provided in Table 5. There were no associations between the level of cognitive change and the likelihood of ever being hospitalized or number of days spent in hospital for psychiatric reasons.

**Table 5:** Hurdle models of the relationship between cognitive change and hospitalization.

	Descriptive	Hurdle model - logistic binary component		Descriptive	Hurdle model – truncated negative binomial component	
	No. (%)	OR (95 % CI)	*p* value	Mean, Median (Range)	IRR (95 % CI)	p value
Days in hospital	57 (79 %)	0.79 (0.39–1.60)	0.513	98.2, 40, (3 - 759)	0.97 (0.62-1.52)	0.905
Number of admissions	57 (79 %)	0.79 (0.39–1.60)	0.513	2.8, 2, (1-13)	1.20 (0.69-2.10)	0.514

### 3.2 Health care usage

Sixty-eight of the 72 eligible patients were followed for approximately 5 years or more. For these individuals, the 12-month prevalence of health care usage in their fifth year after inclusion in the study was summarized and analyzed in relation to cognitive change. In the long-term follow-up sample, 49 individuals (72 %) remained in contact with specialized outpatient psychiatry (at least one visit with a medical doctor). Thirty-four individuals (50 %) had outpatient visits where the main diagnosis was schizophrenia spectrum or other psychotic disorders (ICD-10 codes, F20-F29). In total, 42 individuals (62 %) were still taking antipsychotic medications, based on registered dispensations.

Using logistic regression, we analyzed whether the magnitude of cognitive loss leading up to psychosis could predict subsequent clinical outcomes in the fifth year following inclusion in the study. Cognitive change was not significantly associated with continued contact with outpatient psychiatric services (for any diagnosis); odds ratio (OR) of being a patient at 5 years, per unit decrease in slope-value = 1.27, *p* = 0.471. However, greater cognitive decline was significantly associated with increased likelihood of having an outpatient psychiatry contact for psychotic disorder (OR = 1.89, *p* = 0.046), as well as with purchasing antipsychotic medications (OR = 2.08, *p* = 0.027).

### 3.3 Biological markers

C4A protein levels were not significantly associated with the degree of cognitive change in patients (β = 0.017, *p* = 0.384, *N* = 37). Similarly, there was no significant relationships between genetic risk and cognitive change in patients, either for the intelligence PRS (β = -0.132, *p* = 0.372) or the schizophrenia PRS (β = -0.005, *p* = 0.978), *N* = 45.

### 3.4 Sensitivity analysis

When adjusting for sex, the effect of cognitive change on the likelihood of being a patient in outpatient psychiatry for a psychosis diagnosis was no longer statistically significant (OR = 2.28, *p* = 0.067), although the effect size remained largely unchanged. Cognitive change was still a significant predictor of using antipsychotics in the fifth year after inclusion, even when adjusting for sex (OR = 1.96, *p* = 0.034).

## 4 Discussion

In this study, which combined registry data on school grades as a proxy of premorbid cognitive functioning with results from standardized cognitive tests, we observed a large mean cognitive decline in patients at psychosis onset, accompanied by considerable variability in individual trajectories. The magnitude of cognitive change over time predicted long-term clinical outcome, but was not associated to number of inpatient days, genetic risk, or cerebrospinal fluid levels of C4A.

The average effect associated with illness onset in our sample was large, a loss of over 1.1 population standard deviations, which is in line with previous cross-sectional comparisons with healthy controls (Schaefer et al., 2013; Mesholam-Gately et al., 2009; Lee et al., 2024; Fatouros-Bergman et al., 2014) and longitudinal studies in patients (Mollon and Reichenberg, 2018), but larger than the effect reported in a recent meta-analysis on the subject (Cohen et al., 2025). With regard to inter-individual differences in cognitive decline, the standard deviation among FEP individuals was 0.934, implying a 95 % population range of between -2.95 and +0.70. This finding highlights substantial differences between individuals in the degree of cognitive change from school age to psychosis onset, which to our knowledge has not been quantified in this manner before.

School grades did not differ significantly between groups prior to psychosis onset. This contrasts with previous follow-back studies (Dickson et al., 2020; Fuller et al., 2002; Bilder et al., 2006) that have shown differences in school performance before age 16. However, as effect sizes reported in previous studies were small, our modest sample size may have limited our ability to detect such differences. When examining differences between specific timepoints, there was a general tendency, in both patients and controls (although only statistically significant in controls), for individuals to experience a decline in performance between school years 9 and 12. We believe this pattern is likely explained by the change in comparison population used to derive standardized scores: not all individuals who graduate from year 9 go on to graduate from year 12. Hence, the comparison population only contains those who go on to higher education, while those who do not are overrepresented in the lower ranges of the distribution. In both groups, cognitive performance tended to improve between the baseline assessment at study inclusion and the 1.5 year follow-up. This improvement may, at least in part, reflect practice effects (Watson et al., 2022).

In terms of clinical outcomes, we did not find any significant association between the magnitude of cognitive change and number of admissions or hospital days, adjusted for time at risk. This finding contrasts with previous research examining associations between cognitive function and hospitalization (Kadakia et al., 2022). Several factors may explanation this discrepancy. Hospitalization might be more strongly influenced by a lack of cognitive resources, rather than by relative cognitive decline. Moreover, most FEP individuals in our cohort were recruited from inpatient settings, which is consistent with previous studies in Sweden showing that a majority are admitted to hospital at least once during the first five years of illness (Cullen et al., 2023). As such, the first hospitalization may be less indicative of poor long-term outcome than subsequent hospitalizations. For long-term clinical outcome, the level of cognitive decline was not associated with continued psychiatric care in general; however, it was significantly associated with continued contact for psychotic disorders and with dispensation of antipsychotic medications. This may suggest that those who experience greater cognitive decline are more likely to require long-term treatment specifically for psychosis.

Cognitive change was not significantly associated with PRS of either intelligence or schizophrenia. Given that effect sizes of these genetic risk markers on clinical outcomes are generally very small (Liebers et al., 2016; Mallet et al., 2020; Richards et al., 2019), the lack of correlations in our study may be attributed to limited statistical power due to the small sample size. Notably, prior work has shown significant differences in PRS for cognition and schizophrenia between cognitive trajectory groups in larger samples (Dickinson et al., 2020). It could also be speculated that the relationship between cognitive trajectories and PRS is influenced not only by the relative change from previous levels (which is what we modelled), but also by baseline cognitive ability (which was included in the subgroup analysis implemented in the previous study by Dickinson et al. (2020)). Beyond genetic risk, we hypothesized that C4A levels - a marker related to synaptic pruning - would be associated to the degree of cognitive decline. However, we found no such relationship in our sample. Previous work in this FEP cohort has shown that higher C4A levels predict development of schizophrenia (Gracias et al., 2022), and other studies have linked elevated C4A to worse cognitive performance in larger patient samples of (O'Connell et al., 2022; Donohoe et al., 2018). The absence of a relationship with cognitive change over time suggests that other mechanisms may be involved in the longitudinal course of cognition than those associated with cognitive impairment in established illness.

### 4.1 Limitations

Several important limitations should be acknowledged. Although we aimed to model more precise trajectories of cognitive change, the wide variation in age at psychosis onset made it infeasible to model time in years. Additionally, we were not able to obtain data for all individuals at all timepoints, with particularly limited data availability for year 12 grades and the 1.5-year follow-up. The exploratory nature of the analysis and the modest sample size underscores the for replication in independent samples.

Although not statistically significant, there were baseline differences between groups that may have influenced our results. The control group was more highly educated and included a higher proportion of women than the FEP group. However, despite these group differences we found no differences in school grades prior to psychosis.

The KaSP study was designed specifically to investigate biomarkers of interest at illness onset. This design allows for unique analyses early in the disease course; however, the inclusion of drug-naïve and minimally-treated individuals with FEP means our cohort may not be fully representative of the broader population of psychosis patients. The exclusion of co-occuring substance use disorder further limits the generalizability of our findings. Additionally, inclusion of a broader set of psychotic disorders (rather than just schizophrenia) allows for capturing individuals earlier but may also have increased variability in the data, potentially complicating interpretation.

There are also caveats in the interpretation of clinical outcomes. Firstly, we were unable to control for emigration. Therefore, individuals not appearing in medication and care registries may have emigrated, rather than recovered. Secondly, the medication registry logs dispensations and not prescriptions of drugs. Given the high rate of non-adherence to antipsychotics (Guo et al., 2023), some individuals where clinicians have assessed a need for continued treatment may not have been captured. Finally, outpatient care registries includes only visits with medical doctors in specialized care, which may underestimate the true extent of outpatient service use. Nevertheless, in specialized psychiatric care for psychosis, there is a mandate to provide yearly follow-ups with a medical doctor to all patients, which is why we chose to examine the 12-month period prevalence of outpatient contacts.

## 5 Conclusions

Our results demonstrate substantial inter-individual variability in cognitive change from the premorbid period to after psychosis onset. While the degree of cognitive change was not associated with the selected biological variables, it did predict worse clinical outcomes. Given the exploratory nature of the analysis and the limited sample size, our results require replication in independent cohorts.

### 5.1 Clinical implications

This study demonstrates that individuals with FEP exhibit not only considerable variability in cognitive impairment at illness onset, but also in the degree of cognitive decline relative to pre-morbid cognitive function. This further reinforce the importance of individual neuropsychological assessments, which should not solely rely on population norms to define impairment but also incorporate estimates or measurements of pre-morbid cognitive ability.

## CRediT authorship contribution statement

**Maria Lee:** Writing – review & editing, Writing – original draft, Investigation, Formal analysis, Conceptualization. **Alexis E. Cullen:** Writing – review & editing, Supervision, Methodology, Conceptualization. **Granville J. Matheson:** Writing – review & editing, Visualization, Supervision, Methodology. **Zheng-An Lu:** Methodology, Formal analysis. **Sarah E. Bergen:** Writing – review & editing, Supervision, Methodology. **Carl M. Sellgren:** Writing – review & editing, Resources, Investigation, Funding acquisition. **Sophie Erhardt:** Resources, Funding acquisition. **Helena Fatouros-Bergman:** Writing – review & editing, Investigation, Conceptualization. **Simon Cervenka:** Writing – review & editing, Supervision, Resources, Investigation, Funding acquisition, Conceptualization.

## Code availability

The code for reproducing the analyses and figures in this article are available at https://github.com/MariaLeeR/Cognitive_trajectoriesFEP.

## Funding sources

ML was supported by the Professor Bror Gadelius Memorial Foundation. GJM was supported by the 10.13039/501100004359Swedish Research Council (Grant No. 2020-06356). SE was supported by the 10.13039/501100004359Swedish Research Council (Grant No 2024-02812 & 2017-00875), the Swedish Brain Foundation (SE:FO2023-0333), the 10.13039/501100004047Karolinska Institutet and 10.13039/501100004348Stockholm County Council (Grant No 20190175), the Åhlen foundation, and Torsten Soderberg Foundation. CMS was supported by the 10.13039/501100004359Swedish Research Council (Grant no 2023-02827), 10.13039/501100004047Karolinska Institutet and 10.13039/501100004348Stockholm County Council (Grant no 20190447), The 10.13039/501100007687Swedish Medical Society and Erling Persson Foundation. SC was supported by the 10.13039/501100004359Swedish Research Council (Grant No 523-2014-3467), 10.13039/501100004047Karolinska Institutet and 10.13039/501100004348Stockholm County Council (Grant No 20160328 & 20180487).

## Declaration of competing interest

The authors declare the following financial interests/personal relationships which may be considered as potential competing interests: Alexis E. Cullen reports a relationship with Stratenym Inc. that includes: consulting or advisory. Alexis E. Cullen reports a relationship with Symmetron Ltd. that includes: consulting or advisory. If there are other authors, they declare that they have no known competing financial interests or personal relationships that could have appeared to influence the work reported in this paper.

## Data Availability

Owing to institutional restrictions, the data cannot be shared openly but can instead be made available upon request on a case-by-case basis as allowed by the legislation and ethical permits. Requests for access can be made to the Karolinska Institutet's Research Data Office at rdo@ki.se.

## Appendix A Supplementary data

Supplementary material Image 1
